# Bromide of Ethyl

**Published:** 1880-06

**Authors:** 


### ARTICLE VII.

### *Bromide of Ethyl.*

This new anæsthetic has been recently advanced on the testimony of Drs. Turnbull and Levis, of Philadelphia, who have submitted it to quite an extensive trial, as a substitute for chloroform and ether. It is a transparent, colorless fluid, of a sweetish taste, rather agreeable ethereal odor, and sparingly soluble in water. The agent is, however, in fact, not a new one, having been discovered as far back as 1827, from which time until 1849 it attracted little or no attention. In the latter year Dr. Thomas Nunnelly, of Leeds, England, made some experiments with it on animals. In 1865 he read a paper on the subject before the British Medical Association, but so great was the popularity of chloroform at that time that but little attention was paid to the ethyl bromide. The agent is now, therefore, new only in the sense of the term as applied to many other articles of recent introduction, that is, its properties are but being developed and the profession being familiarized with them.

The claims made for the bromide of ethyl are based on its non-irritant action either on the respiratory or other membrane, it being capable of injection subcutaneously, of application to the conjunctiva or to the means of the ear without exciting inflammation or irritation. It is said, also, to be more rapid of action, more rapidly eliminated, more pleasant of administration than even chloroform, and less apt to produce retching or vomiting. The chief claim made for it, however, is its safety as compared with all other anæsthetics. Safety is the great desideratum of anæsthesia, and it is not strange that the profession should hail an agent whose claims to supply it are so strongly urged. Bromide of ethyl has been employed in Philadelphia in over two hundred cases, not only without untoward result but with happy effects never associated with the administration of any other anæsthetic. Such a record is certainly sufficient to entitle it to sufficient confidence to ensure a more extended trial by the general profession.

But in the midst of the general rejoicing at the securing of the great boon comes a report from New York, which in itself admonishes us to take heed where we stand lest we fall. Chloroform was administered to thousands of cases before a death occurred, and for a long time ether was considered absolutely safe. Bichloride of methylene has been given by Spencer Wells a thousand times without an accident and yet a voice comes up from the profession saying that it is not less dangerous than chloroform. Dr. J. Marion Sims, in the *Medical Record* of April 3rd, reports a case in which he lays the death at the door of bromide of ethyl. The patient was a young woman on whom Battey's operation was performed for the relief of epileptic attacks supposed to be due to a diseased ovary. The operation lasted an hour and a half; the patient recovered speedily from the anæsthesia, but was left with a violent headache and retching, and the vomiting which developed during the administration of the anæsthetic continued unabated. A profuse diarrhœa of a cholerine nature, also set in, the discharges being of a dirty brownish watery nature with tenesmus and mucus, and smelling heavily of the vapor of ethyl. The symptoms continuing delirium supervened and the patient died about twenty-one hours after the operation. A *post mortem*

examination was made and the tissues were found to be strongly impregnated with ethyl; the urine also contained it. The kidneys which to the naked eye looked normal were found under the microscope to contain an increase of interstitial tissue together with signs of catarrhal nephritis. These are the essential facts of the case. Whether the death was caused by the ethyl or by the diarrhœa or cholerine, and if the latter whether it was set up by the ethyl or by three drachms of Rochelle salts given some five hours before the operation, are points which remain unsettled. The case disproves the claim made for ethyl that it is entirely gotten rid of by the lungs, and shows that, as in the case of ether, the kidneys play an important part in the process of elimination. This latter point conceded, it follows that there is danger in the administration of bromide of ethyl in organic disease of the kidney. The danger is similar to that attending the use of ether. In the deaths reported as following the use of ether the kidneys have been found to be affected and the suspicion created that uræmia was an important factor in the causation of death.

Dr. Sims closes the report of his case as follows:

"Dr. Levis has had a larger experience with this anæsthetic than any other surgeon, but his longest operation was but forty minutes, whereas my unfortunate case lasted an hour and a half. It may be that we did not administer the ethyl as skillfully as it should have been done. But I do not think we could have given less and maintained the anæsthetic sufficient for its purpose.

If the operation had been comparatively short—if it had lasted only twenty or thirty minutes—if it had terminated before my patient's system, solids as well as fluids, became thoroughly saturated with it, she would in all probability have recovered; but, as it was, it seemed to kill by supersaturation which could not be eliminated, even with the aid of the whole mucous tract of the alimentary canal to aid the lungs. If mine had been a short operation, I might to-day have been lauding the new anæsthetic as a veritable boon to surgery. But, as it was otherwise, I must raise a warning voice, especially in prolonged operations.

It would be an interesting scientific inquiry to determine if ethyl-poisoning by inhalation produced cholerine with a deeply congested condition of the mucous membrane of the lower portion of the alimentary canal, and also to determine what other pathological changes it might induce.

This could easily be done by experiment on the lower animals, and it is to be hoped that some of our physiological *savans* will soon tell us by such experiments what we have to hope and fear from the long continuance of the bromide of ethyl as an anæsthetic.

The inference that I draw from the facts in the history of this case is that the anæsthetic was the cause of death, while the manner of death may have been by uræmic poisoning. The lesson from this is, never to give bromide of ethyl in prolonged operations, and never to give it where there is organic disease of the kidneys. What, then, shall we give?"

While it would perhaps be scarcely just to hold hydrobromic ether responsible for the death the case is sufficient to dispel the hope that the desideratum of anæsthetic, absolute safety, has been supplied, and to convince the profession that care is no less necessary than in the use of other anæsthetics. The warning comes in season and may be the means of preventing the abuse of the drug to which its pleasant nature and the ease of its administration is prone to tempt.—*Therapeutic Gazette.*

